# Obituary: Professor AB Johan Groeneveld

**DOI:** 10.1186/s13054-016-1542-8

**Published:** 2016-11-10

**Authors:** Johannes P. C. van den Akker, Diederik A. M. P. J. Gommers

**Affiliations:** The Department of Intensive Care, Erasmus Medical Center, ‘s-Gravendijkwal 230, 3015 CE Rotterdam, The Netherlands

**Keywords:** Obituary, Johan Groeneveld, AB Johan Groeneveld

Professor Arie Bastiaan Johan Groeneveld (AB Johan Groeneveld as he styled himself) died after a long and bravely fought battle with cancer on Sunday 18 September 2016, at the age of 60.

He studied medicine at the Vrije Universiteit (VU) of Amsterdam in the Netherlands and specialized in the field of internal medicine at his home university. He was a research fellow in critical care medicine in Chicago under the supervision of Professor Max Harry Weil. Upon returning to Amsterdam, he wrote his PhD thesis on peripheral vascular function during septic shock under the supervision of his mentor and collaborator Professor Bert Thijs. Johan worked in the Department of Intensive Care at the VU Medical Centre from 1978 until 2011. In 2004, he became a professor of experimental intensive care medicine. In 2011, he moved to the Erasmus Medical Centre of the University of Rotterdam and was appointed as a professor of experimental intensive care medicine.

Working with Johan was quite an experience. When he came to Rotterdam, he spent half of his time in the ward with patients and the other half focused on research. In the ward, he was able to process the (seemingly) randomly supplied clinical information within a split second and integrate it into a completely coherent summary of what was wrong with the patient: the diagnosis, the (patho)physiological mechanisms, and possible treatment options together with a list of references from the original literature. When no literature was available, he used logic and drew from an impressive memory filled with all kinds of medical knowledge. He explained his choices, and if no evidence was available, he would say, “I smell printing ink”, meaning that we have to do the research ourselves. This entire process would occur in a couple of minutes. During meetings he always kept everyone intellectually sharp, often just by saying: “I cannot remember any published paper that supports what is being said”, and we all knew he was probably right. Of course we checked for ourselves and we had to admit that his remark was indeed true. It was also commonplace for him to say: “I just submitted a paper about this subject”, which was also true.

He worked with an intensity and speed that was unmatched. An e-mail sent with an attachment consisting of a couple of pages of text was returned within minutes with very specific and pointed comments in concise sentences, although sometimes there would only be key words. Sitting next to him when he was evaluating a draft was the intellectual equivalent of riding a roller-coaster on steroids. The swiftness of reading, absorbing all the words and correcting whole sentences was done with lightning speed. As a reviewer, he was always very quick to respond. There were many times when he provided original ideas and drew conclusions other than those the authors of the draft had reported or had even considered just by quickly looking at the data.

At times, he could be very critical and demanding of himself and others. He was very disciplined and used every minute, and he could become very annoyed when he thought other people were wasting his time. However, most of the time he showed a great sense of humour and had sharp observational powers about people and social interactions using, as he said, his “very sensitive antennae”. When you spoke to him, he looked at you over the golden rim of his glasses with sparkling eyes with a mixture of kindness, fun, and shrewdness.

His office was legendary. Neatly stacked photocopies of articles over a foot high, some very old and yellowed, filling almost every space in the room and occasionally occupying floor space. Despite this volume, he was able to find any document immediately. When people said that some papers he advised to read were slightly older, his reaction was: “if it is older, it does not mean it is bad”. He always went back to the original publications to decide for himself, as he never trusted an interpretation of the content of an original paper that was used as a reference.

For most, he was a mentor and he inspired many people. He spoke with everyone who wanted to see him and had a large number of collaborations. He was also very active in education and spoke at many conferences. He could be a fierce opponent when he used his immense intellectual powers, especially in pro-con debates, which were one of his passions and were great fun and educational to watch.

It was difficult to watch Johan gradually become physically weaker. The last time he came to his office in Rotterdam a little over two weeks before he passed away, he could barely walk or eat. Even in this condition he still wanted to help researchers and took part in discussions with us at the canteen during lunch. We could still see that twinkle in his eyes when he listened or made a comment. Outside his work he loved being with his family, reading, skiing and listening to music. He was an accomplished classical pianist himself. Professionally, scientific research, thinking, discussions, arguing, writing, and editing were like oxygen for him.

He was a great source of inspiration. We are fortunate to have known him and we will miss him.

